# Integrating laboratory medicine with the patient

**DOI:** 10.1186/1878-5085-5-S1-A127

**Published:** 2014-02-11

**Authors:** Ian D Watson

**Affiliations:** 1 European Federation of Clincial Chemistry & Laboratory Medicine, Milano, Italy

## 

The digital age is empowering patients and the paradigm of the patients’ relationship with healthcare delivery is changing. If 70% of patient episodes involve laboratory medicine, then the patient’s access to this and their understanding of its meaning for them needs to be addressed, a potentially significant task. There is a growing awareness that we should empower patients with better access to their tests and to the necessary information and that in addition to support from their physician that Specialists in Laboratory Medicine can enable this agenda. The potential limitations and problems inherent in this approach are as much to do with boundaries and communication methods as they are to do with the recipient’s comprehension, which we would need to understand.

